# Isolation and Identification of Feline Herpesvirus Type 1 from a South China Tiger in China

**DOI:** 10.3390/v6031004

**Published:** 2014-02-28

**Authors:** Heting Sun, Yuanguo Li, Weiyi Jiao, Cunfa Liu, Xiujuan Liu, Haijun Wang, Fuyou Hua, Jianxiu Dong, Shengtao Fan, Zhijun Yu, Yuwei Gao, Xianzhu Xia

**Affiliations:** 1 College of Wildlife Resource, Northeast Forestry University, Haerbin, 150040, China; E-Mail: xiaofengsht@163.com; 2 College of Veterinary Medicine, Gansu Agricultural University, Lanzhou, 730070, China; E-Mail: liyuanguo0520@163.com; 3 Wildlife Rescue and Breeding Centre of Jilin Province, Changchun, 130122, China; E-Mails: jiaoweiyi2013@163.com (W.J.); liucunfa2013@163.com (C.L.); liuxiujuan2013@126.com (X.L.); haijunwang2013@163.com (H.W.); 4 Shenzhen Wildlife Zoo, Shenzhen, 518055, China; E-Mails: huafuyou2013@163.com (F.H.); dongjianxiu2013@163.com (J.D.); 5 Military Veterinary Research Institute of Academy of Military Medical Sciences, Changchun, 130122, China; E-Mails: fst0007@163.com (S.F.); zhijun0215@gmail.com (Z.Y.)

**Keywords:** feline herpesvirus type 1, virus isolation, animal challenge experiment, South China tiger

## Abstract

In 2012, an FHV-1-like virus was isolated from a tiger that presented with clinical signs of sialorrhea, sneezing and purulent rhinorrhea. Isolation was performed with the FK81 cell line, and the virus was identified by PCR, transmission electron microscopy (TEM), and the phylogenetic analysis of the partial thymidine kinase (TK) and glycoprotein B (gB) genes. A total of 253 bp of the TK gene and 566 bp of the gB gene were amplified from the trachea of the tiger by PCR/RT-PCR method. Phylogenetic analysis showed that the isolate belonged to the same cluster with other FHV-1 strains obtained from GenBank. Herpes-like viruses with an envelope and diameters of approximately 200 nm were observed in the culture supernatants of FK81 cells inoculated with samples from the tiger. The FHV-1 infection was confirmed by an animal challenge experiment in a cat model. Our finding extends the host range of FHV-1 and has implications for FHV-1 infection and South China tiger conservation.

## 1. Introduction

Feline herpesvirus type 1 (FHV-1; felid herpesvirus 1 (FeHV-1), family *Herpesviridae*, subfamily *Alphaherpesvirinae*, genus *Varicellovirus*) is an important pathogenic agent that causes feline viral rhinotracheitis, which is a highly infectious upper respiratory tract infection of felids [1]. This infection is often fatal to kittens, but adult cats usually survive and exhibit lifelong latency [2,3]. Since the first strain of FHV-1 was isolated in America, infected felids have been reported in many countries, including Canada, Switzerland, the United Kingdom, Holland, Hungary and Japan [4]. There have been no documented reports on FHV-1 in the past few years, although the distribution in China was confirmed by serological survey and virus isolation in domestic cats [5].

The South China tiger (*Panthera tigris amoyensis*) is a tiger unique to China and is the most endangered tiger subspecies, as free ranging individuals have not been found in its historic distribution areas for many years [6]. To aid in the recovery of wild populations, it is common to re-introduce captive individuals into their native range; however, there are fewer than 120 captive South China tigers in China. Furthermore, only a few captive tigers are suitable for reintroduction, and infectious diseases threaten captive tigers. Any sign of disturbance or trouble with the captive populations, in particular any risk of infectious disease, will greatly concern the stakeholders.

In June 2012, a South China tiger in Shenzhen Wildlife Zoo presented with sneezing, purulent rhinorrhea, which ended with its death, although treatment including antibiotics had been tried. In the present study, we used molecular methods, virus isolation, TEM examination and an animal challenge experiment to diagnose the cause of death of the South China tiger, and for the first time, we confirmed the infection with FHV-1 in the captive tiger population in China.

## 2. Results and Discussion

### 2.1. Results

#### 2.1.1. Preliminary Identification of FHV-1 by Molecular Biological Methods

##### 2.1.1.1. PCR/RT-PCR Assays of Clinical Samples for Three Suspicious Pathogens

The AGE (agarose gel electrophoresis) results showed that a target fragment of 292 bp in length was amplified by PCR/RT-PCR, from DNA/RNA extracted from trachea samples of the dead tiger [7]. As indicated, the tested specimens were positive for FHV-1 but negative for other tested pathogens, including canine/feline distemper virus (CDV/FeDV) and feline calicivirus (FCV).

Benefitting from the clinical diagnosis, the authors were able to narrow the range of the laboratory examinations, and, based on the positive result, the subsequent isolation and identification methods focused on FHV-1.

##### 2.1.1.2. Phylogenetic Analysis Based on Two Cloned Gene Fragments of FHV-1

**Figure 1:**
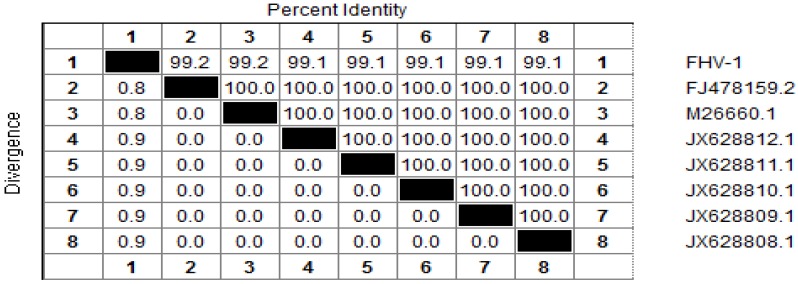
Alignment of the nucleotide sequences of thymidine kinase (TK) gene of Feline herpesvirus type 1 (FHV-1) (**1**), with that of reference strains in GenBank: FJ478159.2 (**2**); M26660.1 (**3**); JX628812.1 (**4**); JX628811.1 (**5**); JX628810.1 (**6**); JX628809.1 (**7**); JX628808.1 (**8**). Nucleotide identity (%) in upper triangle.

The glycoprotein B (gB) gene and thymidine kinase (TK) gene have been selected for the study of molecular phylogeny [7,8]. A 253 bp sequence was obtained, and alignment analysis determined that the TK gene cloned in this study shared a high identity (from 99% to 100%) with that of other FHV-1 isolates (Figure 1). A 566 bp fragment of the gB (glycoprotein B) gene was also cloned, and was found to share 100% identity with that of other FHV-1 isolates. Therefore, its phylogenetic tree was omitted here. The two sequences have been deposited in Genbank whose accession numbers are ** and **, for TK gene and gB gene fragment separately.

**Figure 2:**
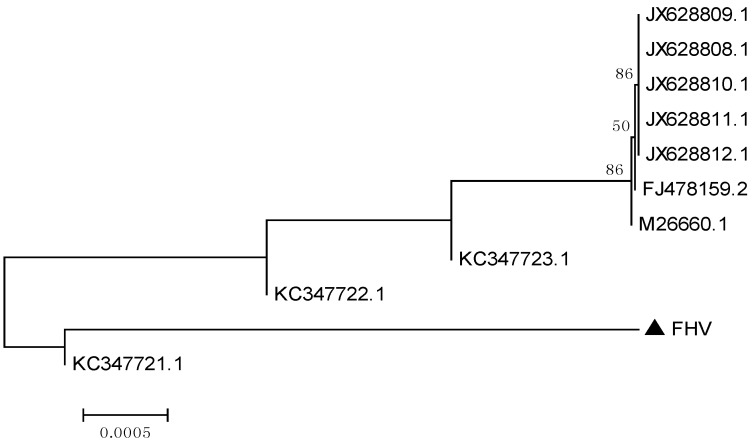
Phylogenetic tree based on the nucleotide sequences of the TK gene. The tree was constructed with MEGA [10]. The isolate of this study is indicated by a solid triangle.

The phylogenetic tree based on the TK gene sequences showed that the isolate investigated in this study, was closely related to the ten isolates of FHV-1 (Figure 2), a result consistent with the alignment analysis.

All isolates of FeHV-1 appear to be relatively similar, as they antigenically belong to one serotype [9]. The TK gene is a conserved gene, and the target fragment is located in its highly conserved region. Therefore, the above results are strong evidence for the presence of FHV-1.

#### 2.1.2. Virus Isolation and TEM Examination of the Cell Cultures

**Figure 3:**
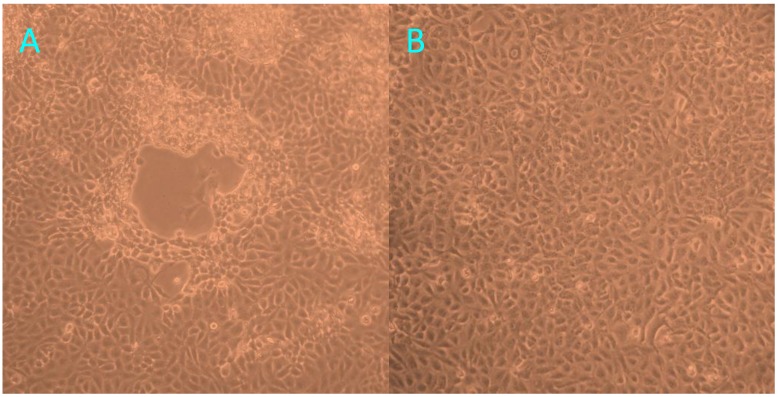
The cytopathic effect observed in FK81 cells inoculated with trachea homogenates from the tiger positive for FHV-1: 72 h post-inoculation (**A**); Normal Fk81cell lines (**B**).

At 72 h post-inoculation with trachea homogenates from the tiger positive for FHV-1, FK81 cell cultures showed a distinct cytopathic effect (CPE), characterized by cell rounding, pyknosis, ‘Fleece-Pulling’ to the thyrsoid and degeneration of the cell monolayer (Figure 3). These effects were also observed in two additional continuous cultures. PCR/RT-PCR revealed that the supernatants of all three cultures were positive only for FHV-1. The positive PCR products of TK gene of FHV-1 from trachea homogenates and cell cultures were identified by sequence analysis. The 253 bp sequence of TK gene from FHV-1 from trachea homogenates shared 100% homology with FHV-1 isolates.

**Figure 4:**
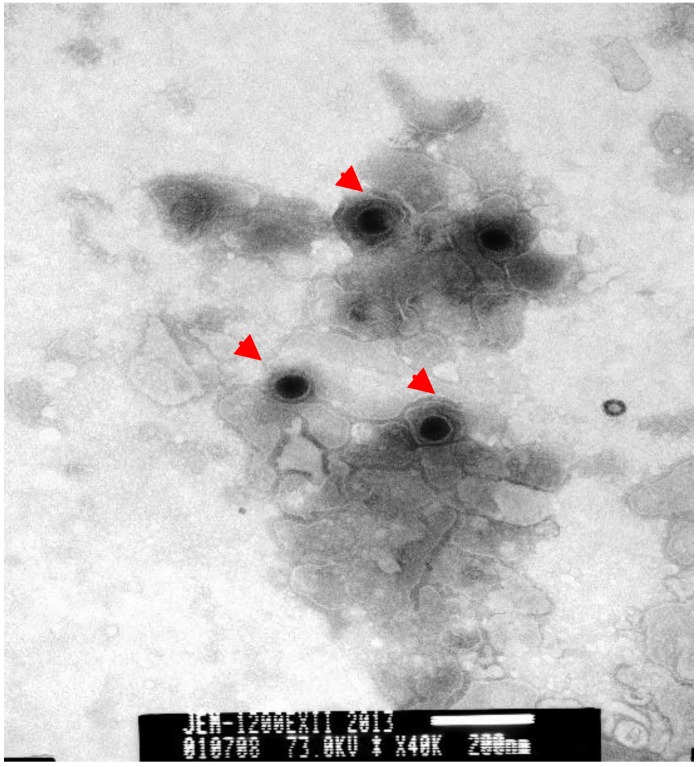
Negative-stained electron micrograph of FHV-1 particles. The electron micrograph of cell cultures (Magnification ×40,000) revealed circular or oval herpesvirus-like particles that appeared as spheres and ellipsoids with diameters ranging from 150 to 200 nm (as arrow indicated). Size bars indicate 200 nm.

TEM of negatively stained samples revealed medium-sized particles with a diameter of approximately 200 nm, an envelope and an electro-dense core (Figure 4), which are characteristics typical of FHV-1.

#### 2.1.3. Cat Challenge Test with the Virus Isolate

##### 2.1.3.1. Clinical Symptoms of the Challenged Cats

**Figure 5:**
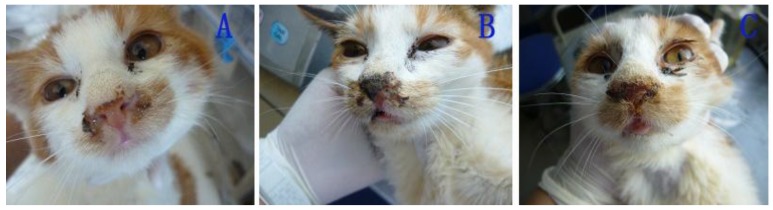
Nasal signs of cats infected with FHV-1: secretion of thin nasal discharge (**A**); purulent secretion (**B**); blocked nostrils (**C**).

After 6 d post-inoculation, the cats in the IG (inoculated group) exhibited sneezing and increasing palpebral secretion (Figure 5A,B). Subsequently, their nostrils were completely blocked with purulent secretions resulting from thin nasal discharge (Figure 5C). After the 18th day, the cats recovered completely and exhibited no signs of infection. Similar signs and similar clinical courses were observed in the cats in the EG (exposed group) and in the cat of the control group, but the initial clinical signs manifested on the 11th day and the 16th day post-inoculation, respectively.

**Figure 6:**
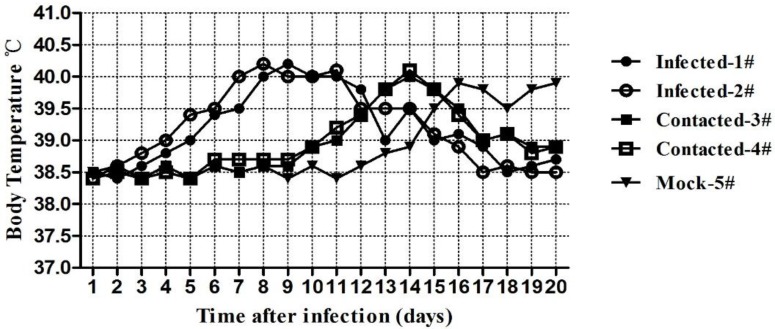
Temperature changes of cats infected with FHV-1.

The changes in the cat’s body temperatures generated a curve with a single peak. For the IG group cats, pyrexia began at day 4 post-inoculation and continued for 14 days, with a peak of 40.2 °C. For the EG group and control group cats, fever was detected on the 10th day and the 13th day, respectively (Figure 6).

##### 2.1.3.2. Virus Shedding in the Infected Cats

For the IG group cats, positive results appeared on the 6th day post-inoculation. PCR was used to detect FHV-1 DNA extracted from the specimens, which included nose washing fluid, eye swabs and swallow swabs. Positive results were found on the 10th day and 16th day for the EG group cats and the control cat, respectively (Table 1). These results revealed a baseline of virus shedding in the infected cats after they were inoculated or were exposed to FHV-1.

**Table 1:** Results of viral DNA detected in samples collected from cats. A minus sign was used to represent negative PCR results, and a plus sign was used for positive results. The samples were collected on even days.

Cat No.	Days post-inoculation									
	0	4	6	8	10	12	14	16	18	20
1#	−	−	+	+	+	+	+	+	+	+
2#	−	−	+	+	+	+	+	+	+	+
3#	−	−	−	−	+	+	+	+	+	+
4#	−	−	−	−	+	+	+	+	+	+
5#	−	−	−	−	−	−	−	+	+	+

The incubation period of FHV-1 infection as indicated by this challenge test is approximately 6 days, which is consistent with that reported previously [11]. Additionally, clinical signs, including pyrexia, inappetence, and serous ocular and nasal discharge, were observed in the cats [12], and a long period of virus shedding was observed [13].

### 2.2. Discussion

Although most FHV-1 strains produce a relatively uniform disease seen primarily in the respiratory tract, pancreatitis and generalized disease may be seen occasionally in debilitated animals or in neonatal kittens [14,15,16]. The FHV-1 described in this study, was isolated from a captive tiger that exhibited respiratory signs that were suspected to be due to rhinotracheitis. By PCR/RT-PCR, the only virus detected in the trachea homogenates was FHV-1, which was confirmed afterwards by virus isolation, the TEM examination of cell cultures showing CPE, and a challenge experiment in cats. Furthermore, the full genome of the virus isolated in cell culture is being sequenced, and the obtained sequences are 90%–100% homologous with that of PCR products either for TK gene or for gB gene. Challenged cats exhibited uniform clinical symptoms and shed FHV-1 virus. Other agents causing respiratory disease were excluded as possible pathogens.

The tiger was born in Shenzhen Wildlife Zoo, and had chances to contact stray cats and other felids during its lifetime. On its infection, the authors supposed two possible sources. One was that the tiger got the virus in captivity perhaps due to stray cats in the zoo, because FHV-1 was shed in ocular, nasal, and oral secretions, and transmission was primarily by direct contact with an infected cat. The other was an endogenous infection originating from a latent viral infection. As with other alphaherpesviruses, latency is common, and periodic viral reactivation is sometimes associated with the reoccurrence of clinical signs [17]. To validate the first supposition, a retrospective investigation was conducted after the virus isolation and identification. Although negative PCR results were found for the swab samples collected from stray cats and captive leopards and tigers, the contagious source could not be easily obviated. It is very much regrettable that neither prior sera nor post mortem sera were stored enabling the seroconversion analysis, making our second supposition to be theoretic.

FHV-1 is relatively fragile in the environment and is highly susceptible to the effects of common disinfectants [18]. Even though negative PCR results of the retrospective survey indicated that widespread infection was unlikely to occur in the zoo, serological data of felid animals would be obtained in the next research to assess the epidemic risk for tigers better and more precisely.

Currently available evidence indicates that the host range of FHV-1 includes several members of the Felid such as cheetahs [19], lions [20], and wild and domestic cats [21], but to our knowledge, FHV-1 infection has not been reported in tigers previously. This report describes the first occurrence of FHV-1 in a South China tiger in China and extends the species range, confirming that this virus poses a risk to this species.

## 3. Experimental Section

### 3.1. Case Description and Sample Collection

Because of the clinical signs, the tiger was initially diagnosed with rhinotracheitis, and only trachea samples were obtained for laboratory examination after it died. The viral pathogen of felidae include canine/feline distemper virus (CDV/FeDV), feline calicivirus (FCV) and FHV-1, we first considered these three agents. Polymerase chain reaction (PCR) and reverse transcription polymerase chain reaction (RT-PCR) were used to detect these viruses. The PCR/RT-PCR results indicated the presence of FHV-1.

### 3.2. Specimen Treatment

The weighted trachea specimen was ground (parameter: 30 tps) into a homogenate with a beveller (type: QIAGEN, Spoorstraat, Netherlands), and then the products were ground for 3 minutes (parameter: 30 tps) again, to which serum-free MEM was added. The supernatant obtained from a 5-minute centrifugation (at 4 °C and 5000 rpm) was fractionated into two centrifuge tubes for later tests.

### 3.3. Molecular Identification

#### 3.3.1. PCR/RT-PCR Assays

Viral genomic RNA and DNA were extracted from the supernatant obtained in Section 3.2 using a Multisource Genomic DNA/RNA Miniprep Kit (Axygen, Hangzhou, China), subjected to RT-PCR or PCR and screened for the three potential pathogens (Table 2).

**Table 2:** Oligonucleotide primers, reaction systems and conditions used for the amplification of the target genes of the three suspected viruses.

Virus	Target gene	Primer sequence 5'-3'	Amplified fragment	Reaction systems (50 μL)	Conditions for PCR	Reference
FHV-1	TK-F TK-R	GACGTGGTGAATTATCAGC CAACTAGATTTCCACCAGGA	292 bp	10× ExTaq Buffer, 5 μLdNTP (2.5 mmol/L), 4 μL F/R primer, 1 μL Template, 2 μL ExTaq polymerase, 1 μL ddH_2_O, 36 μL	Fore- denaturalization: 94 °C, 5 min. Denaturalization: 94 °C, 30 s. Anneal: 56 °C, 30 s. Extension: 72 °C, 30 s. 30 cycles, extension: 72 °C, 10 min.	[7]
FCV	Cali1 Cali2	AACCTGCGCTAACGTGCTTA CAGTGACAATACACCCAGAAG	924 bp	10× ExTaq Buffer, 5 μL dNTP (2.5 mmol/L), 4 μL A/B primer, 1 μL Template, 2 μL ExTaq polymerase, 1 μL ddH_2_O, 36 μL	Fore- denaturalization: 94 °C, 2 min. Denaturalization: 94 °C, 60 s. Anneal: 56 °C, 45 s. Extension: 72 °C, 60 s. 35 cycles, extension: 72 °C, 7 min.	[22]
CDV	NfpNrp	GCTGGTTGGAGAATAAGG CCAACTCCCATAGCATAA	586 bp	10× ExTaq Buffer, 5 μL dNTP (2.5 mmol/L), 4 μL A/B primer, 1 μL Template, 2 μL ExTaq polymerase, 1 μL ddH_2_O, 36 μL	Fore- denaturalization: 94 °C, 3 min. Denaturalization: 94 °C, 30 s. Anneal: 60 °C, 30 s. Extension: 72 °C, 30 s. 33 cycles, extension: 72 °C, 10 min.	[23]

#### 3.3.2. Gene Cloning and Sequence Analysis

The target fragments observed by agarose gel electrophoresis (AGE) were extracted by a DNA Gel Extraction Kit (Axygen, Hangzhou, China), and were cloned into a pGEM-T Easy vector with routine methods [24]. The sequencing of the cloned plasmid was performed by BGI Sequencing, and the results were submitted to GenBank for alignment analysis. The phylogenetic tree was constructed using MEGA-5 software [10].

### 3.4. Virus Culture

Briefly, FK-81 cell monolayers (from Toronto University of Canada and stored in this lab) in Costar flasks were inoculated with 1 mL supernatant (obtained in Section 2.1) treated with antibiotics at final concentration 100 U/mL. After gentle rotation, the flasks were incubated for 1 h at 37 °C in 5% CO_2_ to allow for attachment; then, the supernatants were removed, and the monolayers were washed three times with MEM without FBS. After washing, 7 mL DMEM with 2% FBS was added, and the flasks were then incubated for 3 days at 37 °C in 5% CO_2_. If the cells did not exhibit a cytopathic effect (CPE) by the fourth day, the incubated monolayers were subjected to another two passages, and the culture supernatant (500 μL) was collected for PCR/RT-PCR. The monolayers were not discarded, unless the PCR/RT-PCR results were negative and no CPE was observed in the third FK-81 passage. If 80% of cells exhibited CPE and the PCR/RT-PCR results were positive, the cells were frozen for further analysis.

### 3.5. Electron Microscopy Examination

The cultured cells were harvested and fixed in 2.5% glutaraldehyde for transmission electron microscopy (TEM) examination (JEM-1200, Tokyo, Japan).

### 3.6. Animal Challenge Test

Five healthy domestic cats free of FHV-1 and FHV-1 antibodies, aged 3 months old and weighing from 1.8 kg to 2.0 kg, were divided into three groups. Because the transmission of FHV-1 is largely by direct contact with an infected animal, the cage for cats No. 1 and No. 2, which made up the IG (inoculated group), was placed at the bottom, the cage for cats No. 3 and No. 4, which made up the EG (exposure group), was placed in the middle, and the cage for cat No. 5, the control cat, was placed at the top. Under anesthesia, the two cats of the IG were inoculated by intranasal and ocular routes with 0.5 mL sample/cat containing 10^6^ TCID_50_ of the SZ12 strain, and the other cats were mock-inoculated with 0.5 mL PBS/cat. Each cat’s temperature was taken, and clinical signs were observed daily. From the fourth day post-inoculation, nose, eye and throat secretions were collected for PCR to detect FHV-1 infection.

## 4. Conclusions

In this study, the authors described the first occurrence of feline herpesvirus type 1 (FHV-1) in a South China tiger in China. The TK gene sequences of the FHV-1 isolate were highly similar to those of other strains. Challenge experiments found that the isolate caused typical clinical signs, a long period of virus shedding and efficient transmission in cats. The study expands the species range for FHV-1 infection and provides new epidemiologic data.
